# The relationships among nature connectedness, climate anxiety, climate action, climate knowledge, and mental health

**DOI:** 10.3389/fpsyg.2023.1241400

**Published:** 2023-11-15

**Authors:** Emily E. Thomson, Sean P. Roach

**Affiliations:** Department of Psychology, University of New Brunswick Saint John, Saint John, NB, Canada

**Keywords:** climate change, mental health, climate anxiety, nature connectedness, pro-environmental behavior, climate knowledge

## Abstract

**Introduction:**

Climate change is a source of global concern that has both direct and general impacts on mental health. A recent study conducted following severe bushfires in Australia demonstrated relationships among nature connectedness, climate action, climate worry, and mental health; for example, nature connectedness was associated with climate worry, which in turn was associated with psychological distress.

**Methods:**

The present study sought to replicate those findings while building on them in two important ways: on those findings in two ways: first, test similar relationships in a different geographical context that has been mostly spared from direct impacts by acute climate events; second, we take into consideration an additional factor, climate knowledge, which has been linked to relevant factors such as climate anxiety.

**Results:**

The results of a survey completed by 327 adults revealed a similar relationship between nature connectedness and climate anxiety, and between that and psychological distress. Further mirroring those previous findings, nature connectedness was associated with both individual and collective climate action, but the relationships between them and psychological distress differed.

**Discussion:**

The proposed model was a better fit to the collected data among those with high levels of climate change knowledge than those with low levels, suggesting that such knowledge influences how the above factors relate to each other.

## 1. Introduction

A recent study by Curll et al. (2022), conducted in Australia following a historically bad bushfire season, explored the ways in which nature connectedness, pro-environmental behaviors, and various facets of mental health (climate worry and psychological distress) are related to each other in the context of acute climatic events. They found that nature connectedness was positively associated with both climate worry and pro-environmental behavior in the forms of both individual and collective action; climate worry and collective action were, in turn, linked to psychological distress. The present study sought to replicate those findings, with two important differences: (1) It was conducted in a geographical location that has been much less impacted by severe weather events; and, (2) It included an additional construct, climate knowledge, that may be important to understanding the above-mentioned relationships.

Climate change represents a grave threat to humans on every scale, from individual wellbeing to the maintenance of society to the planetary health that underlies the ecosystems that sustain us (IPCC, 2022). Along with the well-documented impacts of climate change upon physical health (Watts, 2020), recent research has provided an increasingly clear picture of its negative effects on our mental health. Acute climate change-related weather events such as heat waves, wildfires, and flooding have been linked to poor mental health including elevated rates of depression, anxiety, and post-traumatic stress disorder (Obradovich et al., 2018; Cianconi et al., 2020; Cruz et al., 2020).

Recent findings have made it clear that the psychological effects related to climate change are not just limited to acute events but rather are global in scale. A recent ten-nation study found that 59% of respondents were very or extremely worried about climate change; negative emotions such as sadness and guilt were each reported by over half of the respondents in relation to climate change (Hickman et al., 2021). Highlighting the impacts of awareness and anticipation, a recent longitudinal study by McBride et al. (2021) found that individuals’ concerns about climate change at one time point predicted their levels of psychological distress a year later. Overall, it appears that climate change negatively impacts mental health through both direct and indirect mechanisms (Berry et al., 2010; Doherty and Clayton, 2011).

One relatively new concept that might enrich our understanding of such negative effects of climate change is climate change anxiety (Clayton, 2020; Clayton and Karazsia, 2020), which has emerged in parallel with some other, similar ideas such as eco-anxiety (Coffey et al., 2021) and ecological stress (Helm et al., 2018). As conceptualized by Clayton and Karazsia (2020), climate change anxiety can be distinguished from concern or worry about climate change by its impacts on everyday life, which may be cognitive-emotional (e.g., difficulty concentrating) or functional (e.g., neglecting other facets of life). Climate change anxiety is positively associated with worry and concern related to climate change (Whitmarsh et al., 2022; Tam et al., 2023) but, in keeping with its heightened impacts, also less prevalent (Whitmarsh et al., 2022).

A growing number of studies have associated climate change anxiety with poor mental health. For example, Reyes et al. (2021) found climate change anxiety to be a significant predictor of psychological distress. To some degree, findings are mixed: climate change anxiety has been linked to generalized anxiety in some (Schwartz et al., 2022; Whitmarsh et al., 2022) but not all cases (Mouguiama-Daouda et al., 2022). Depression has also been linked to climate change anxiety (Mouguiama-Daouda et al., 2022), although in one case depression was associated with cognitive-emotional but not functional impairment (Schwartz et al., 2022). It may also contribute to the effects of acute climate events: scores on the Climate Change Anxiety Scale in a western Canadian population were significantly higher following a 2021 heat dome than they had been prior to that event (Bratu et al., 2022).

Given the well-accepted contribution of human activities to climate change, a central research focus has been to understand human willingness to change alter problematic behaviors and engage in action with the potential to slow or reverse its progress. Such pro-environmental behaviors can be divided two categories based on their scope: individual action involves making environmentally friendly purchasing choices, using less gas or electricity at home, etc., while collective action involves behaviors like basing voting decisions upon climate change positions and expressing views on climate change to others (Walker et al., 2015; Stanley et al., 2021). In understanding how they relate to mental health in the context of climate change, it is notable that these two types of action differ in terms of their associated emotions. For example, high subjective wellbeing is more strongly associated with individual than collective climate action. In terms of motivations, engagement in individual climate action has been linked to positive affect (Coelho et al., 2017), whereas collective action appears to be driven by negative emotions such as anger and guilt (Mallett, 2012; Harth et al., 2013).

Researchers have identified several predictors of pro-environmental behavior, including personality (Soutter et al., 2020) and environmental knowledge (Tamar et al., 2021). Climate change appears represent another predictor: it has been positively linked to pro-environmental behavior in several studies (Wullenkord et al., 2021; Heeren et al., 2022), though in other cases that association was either absent (Clayton and Karazsia, 2020) or present for some behaviors but not others (Whitmarsh et al., 2022).

An additional factor that has recently been studied in relation to many of the above factors, in the context of climate change, is nature connectedness, an individual’s sense of emotional and cognitive connection to the natural world (Mayer and Frantz, 2004). Not surprisingly, those that feel more connected to nature engage in more pro-environmental behaviors (Martin et al., 2020; Whitburn et al., 2020). Nature connectedness has in most cases exhibited a positive association with mental health: across many studies, it has been associated with greater well-being, whether hedonic (Capaldi et al., 2014) or eudaimonic (Pritchard et al., 2020), and lower depression, anxiety, and stress (Richardson, 2019; Bakir-Demir et al., 2021). Nonetheless, there is some evidence that nature connectedness may instead be associated with worse mental health in the context of acute climate crises (Dean and Green, 2018; Curll et al., 2022).

Overall, while many studies have taken into consideration one or a few of the above factors related to climate change, it is not clear how they all fit together. Recently, Curll et al. (2022) proposed and tested a path model relating nature connectedness to climate worry, climate action (individual and collective), and psychological distress (depression, anxiety, and stress). Their results revealed that nature connectedness was associated with pro-environmental behaviors in the forms of both individual and collective action, as well as climate worry (and climate worry was positively associated with each type of climate action). In turn, climate worry and collective action were positively linked to psychological distress, whereas collective action was negatively associated with it.

While Curll et al.’s (2022) findings provide valuable insights into the relationships among nature connectedness, mental health, and climate action, it is important to note that it was done in the context of an acute climate crisis. Considering that factors such as climate anxiety vary substantially across populations (Tam et al., 2023) and can change within populations following climate events (Bratu et al., 2022), it is unclear whether these relationships would be similar in other contexts including the absence of such acute circumstances. Thus, one objective of the present study is to assess a model similar to that proposed by Curll et al. (2022) in a different location and out of that type of context. In the present study, data collection took place in Canada’s Maritime provinces, a region that has occasionally experienced seasonal flooding in some areas and strong weather associated with passing hurricanes but otherwise has not experienced extreme weather events (e.g., widespread flooding, wildfires) to the same degree as other regions, such as Australia or western Canada.

Based on documented changes following climate events (Bratu et al., 2022), it was hypothesized that climate change anxiety would be lower in this less affected population. We also hypothesized, based on the global nature of climate change impacts (Hickman et al., 2021; Ogunbode et al., 2022), the proposed path model would be an overall good fit to the data, with the possibility of differences in some relationships compared to the findings of Curll et al. (2022). For example, based on geographic comparisons among countries that varied in vulnerability to climate impacts (Tam et al., 2023), we expected climate anxiety and climate action to be more strongly linked here compared to Curll et al.’s (2022) findings.

Curll et al. (2022) also suggested that future studies consider additional variables that might influence the relationships in question. One such factor is climate change knowledge, which includes knowledge about topics such as temperature changes and greenhouse gases, as well as the meaning of ‘climate’ itself (Reynolds et al., 2010; Tobler et al., 2012). Such knowledge is thought to be a critical factor in individuals’ overall understanding and perception of climate change as an important global topic, as well engagement related to the issue (Wolf and Moser, 2011; Shi et al., 2016); especially considering the high prevalence of misinformation on the topic (Lewandowsky, 2021). Taking climate knowledge into consideration alongside the other variables measured by Curll et al. (2022) has the potential to enrich our understanding of how nature connectedness, climate action, and mental health relate, especially considering that climate knowledge has previously been linked to facets of their model. For example, climate knowledge is positively associated with pro-environmental behaviors (Vicente-Molina et al., 2013) and negatively related to climate anxiety (Zacher and Rudolph, 2023). Nonetheless, the role of climate knowledge in the climate context is likely complex given the possibility that individuals employ defense mechanisms to protect against negative emotional responses to climate change information, which in turn may influence their behavior (Moser, 2007).

Thus, the second objective of the present study is to examine how climate anxiety influences the above-described relationships. Although Curll et al. (2022) did not measure climate or environmental knowledge, it was likely high considering that such topics were at the forefront in that context. Thus, we hypothesized that the proposed model would be a better fit in those with high climate knowledge.

## 2. Methods

### 2.1. Study design

In keeping with the present study’s objectives to expand upon Curll et al.’s (2022) findings regarding the interrelationships among nature connectedness, climate action, and mental health, the same array of variables was measured, with two exceptions. One was the addition of a measure of climate knowledge and the other was replacement of climate worry to climate change anxiety, a distinct but related construct. This switch was inspired by the rapidly growing body of knowledge linking climate change anxiety to the other factors (e.g., climate action) included here.

### 2.2. Participants

Data was collected from a total of 327 individuals during the period of February–April, 2023. Participants were recruited from undergraduate classes at the University of New Brunswick Saint John in Saint John, New Brunswick Canada (*n* = 225); participants were also recruited through social media (*n* = 102) by advertising on Facebook groups and subreddits associated with the University of New Brunswick and the Maritime provinces (e.g., r/SaintJohn). Within the total sample of 327 participants, 236 identified as female, 80 identified as male, and 11 identified as gender variant/non-conforming. The mean age of participants was 24.30 years (SD = 9.19), ranging from 18 to 68.

### 2.3. Measures

#### 2.3.1. Nature connectedness

Nature connectedness was measured using the Connectedness to Nature Scale (CNS) (Mayer and Frantz, 2004). This scale consists of 14 items (e.g., “I often feel part of the web of life”) measured on a 5-point Likert scale ranging from 1 (“Strongly disagree”) to 5 (“Strongly agree”). Responses were averaged to generate a total score, with higher scores representing greater nature connectedness. The scale exhibited high internal reliability (α *=* 0.83).

#### 2.3.2. Climate change anxiety

Climate change anxiety was measured using the 13-item Climate Change Anxiety Scale (Clayton and Karazsia, 2020). Participants responded to items (e.g., “I write down my thoughts about climate change and analyze them”) using a 5-point Likert scale ranging from 1 (“Strongly disagree”) to 5 (“Strongly agree”). Responses on all items were averaged to generate a total score; responses were also averaged to generate scores for cognitive-emotional impairment (items 1–8) and functional impairment (items 9–13), following the two-factor model established by Clayton and Karazsia (2020). The scale’s reliability was high (α = 0.93) in the present study.

#### 2.3.3. Individual and collective climate action

Pro-environmental behaviors in the forms of individual and collective climate action were measured using a 16-item scale (Stanley et al., 2021) that asked participants the degree to which they engaged in such behaviors on a visual scale ranging from 0 (“Never”) to 100 (“At every opportunity”). Eight of the items asked about collective action (e.g., “Written a letter to a member of parliament”) and eight asked about individual action (e.g., “Tried to fix things rather than replace them”); for each, individual responses to generate a total score. Both individual (α = 0.77) and collective action (α = 0.86) exhibited acceptable internal reliability.

#### 2.3.4. Climate knowledge

The Climate Change Assessment Measure (Bodzin and Fu, 2014) was used to measure objective climate knowledge. This questionnaire consists of 28 multiple choice questions assessing general climate and climate change knowledge. Higher scores indicate more climate and climate change knowledge. This questionnaire exhibited acceptable reliability (α = 0.79) in the current study.

#### 2.3.5. Psychological distress (depression, anxiety, and stress)

To measure psychological distress, the 21-item Depression Anxiety Stress Scale (DASS-21; Lovibond and Lovibond, 1995) was used. It consists of subscales for depression, anxiety, and stress, each of which is measured using seven items. Participants were asked to rate the relevance of each statement (e.g., “I found it hard to wind down”) to themselves from 0 (“Does not apply to me at all”) to 3 (“Applies to me very much or most of the time”). Responses were summed to for each subscale, such that scores ranged from 0 to 21 for each variable. The internal reliability scores for depression (α = 0.91), anxiety (α = 0.86), and stress (α = 0.89) were all high in the present study.

### 2.4. Procedure

The study was reviewed and approved by the Research Ethics Board at University of New Brunswick Saint John. After being directed to an online survey platform (Qualtrics) and providing consent, participants provided demographic information and were presented with a randomized package of questionnaires. Data collection occurred between February 15 and April 17, 2023.

### 2.5. Statistical analysis

Analyses were done using the Statistical Package for the Social Sciences software version 25 (SPSS 25) and R software (R Core Team, 2022). Of the 346 participants that completed the survey, 19 were removed for incomplete responses, leaving a final sample size of 327. There were no univariate outliers observed, and using Mahalanobis distances, it was determined that there were also no multivariate outliers.

Bivariate correlation analyses were used to examine zero-order associations among variables. In keeping with the objective of comparing relationships among the measured variables to those seen by Curll et al. (2022), the same hypothesized model (with the replacement of climate worry with climate change anxiety) was tested using path analysis, a form of structural equation modeling. This was conducted using the *levaan* package (Rosseel, 2012) in R for each of the three outcome variables (depression, anxiety, and stress). The fit of each model was assessed using the following indices: comparative fit index (CFI), Tucker Lewis index (TLI) and standardized root mean square residual (SRMR) values, root mean square area of approximation (RMSEA). The criteria for an adequate fit of the model to the data were values greater than 0.90 for CFI and TLI, and values less than 0.08 for SRMR and RMSEA (Kline, 2015).

To test the role of climate knowledge, participants were divided into two groups with the goal of creating two roughly equal sized groups with which to conduct separate path analyses: those that scored at or above 60% on the Climate Change Knowledge assessment were placed in the high climate knowledge group (*n* = 185) and those that scored less than 60% were placed in the low climate knowledge group (*n* = 161). The above-described path analyses were then run separately for the high and low climate knowledge groups with regard to each of the three psychological distress variables. Specific bivariate relationships within the model were compared between the high and low climate knowledge groups using the R package *cocor* (Diedenhofen and Musch, 2015) to statistically compare pairs of correlations. In addition, climate knowledge, treated as a continuous variable, was tested as a possible moderator for each of the bivariate relationships within the model using Hayes Process macro (model 1; Hayes, 2018) in SPSS. A moderation effect was concluded based on a statistically significant interaction (*p* < 0.05) between the focal predictor variable and climate knowledge.

## 3. Results

### 3.1. Descriptives

Descriptive statistics are shown in Table 1. Several of the nature and climate variables differed as a function of gender and/or age. Females exhibited greater nature connectedness, more engagement in individual action, and higher anxiety scores; they also reported greater cognitive-emotional impairment related to climate change anxiety. Males scored significantly higher on the climate knowledge quiz. Age was associated with some of the climate-related variables: older participants reported greater climate knowledge and more engagement in both types of pro-environmental behaviors, as well as more anxiety (Table 2).

**Table 1:** Descriptive statistics (mean and standard deviation) and gender differences (results of unpaired *t*-tests).

Variable	Full sample	Females	Males	Gender comparison
Nature connectedness	3.46 (0.62)	3.51 (0.61)	3.29 (0.58)	*t* = 2.686, *p* = 0.008
Climate change anxiety	1.94 (0.83)	1.99 (0.84)	1.79 (0.81)	*t* = 1.861, *p* = 0.064
Cognitive-emotional	2.01 (0.85)	2.07 (0.87)	1.83 (0.80)	*t* = 2.188, *p* = 0.029
Functional	1.83 (0.89)	1.86 (0.89)	1.72 (0.91)	*t* = 1.166, *p* = 0.245
Individual action	48.10 (18.48)	48.30 (17.64)	46.72 (20.13)	*t* = 0.641, *p* = 0.008
Collective action	24.91 (20.94)	25.57 (20.69)	20.76 (21.83)	*t* = 1.736, *p* = 0.083
Climate knowledge	0.58 (0.17)	0.55 (0.17)	0.64 (0.17)	*t* = −4.199, *p* < 0.001
Depression	6.72 (5.41)	6.47 (5.24)	6.61 (5.30)	*t* = −0.195, *p* = 0.846
Anxiety	6.26 (5.02)	6.69 (5.06)	4.92 (4.57)	*t* = 2.706, *p* = 0.007
Stress	8.23 (5.19)	8.67 (5.24)	6.82 (4.81)	*t* = 2.727, *p* = 0.007

**Table 2:** Bivariate correlations among study variables.

Variable	1.	2.	3.	4.	5.	6.	7.	8.	9.	10.	11.
1. Age	–										
2. Nature connectedness	0.15^*^	–									
3. Climate change anxiety	0.05	0.22^**^	–								
4. Cognitive-emotional	0.04	0.25^**^	0.97^**^	–							
5. Functional	0.06	0.16^**^	0.94^**^	0.83^**^	–						
6. Individual action	0.22^**^	0.34^**^	0.13^*^	0.14^*^	0.10	–					
7. Collective action	0.15^**^	0.37^**^	0.45^**^	0.47^**^	0.38^*^*	0.49^**^	–				
8. Climate knowledge	0.26^**^	0.15^**^	−0.22^**^	−0.18^**^	−0.25^**^	0.09	0.11	–			
9. Depression	0.04	0.11^*^	0.46^**^	0.46^**^	0.41^**^	−0.02	0.29^**^	−0.09	–		
10. Anxiety	0.17^**^	0.08	0.39^**^	0.41^**^	0.32^**^	−0.04	0.27^**^	−0.19^**^	0.63^**^	–	
11. Stress	−0.03	0.12^*^	0.39^**^	0.42^**^	0.32^**^	−0.04	0.27^**^	−0.11	0.65^**^	0.71^**^	–

^*^p < 0.05, ^**^p < 0.001.

### 3.2. Bivariate correlations

Bivariate correlations among the study’s continuous variables are shown in Table 2. The three psychological distress variables and climate change anxiety were all highly correlated with each other. Nature connectedness was positively associated with most climate-related variables, including climate change anxiety (*r* = 0.22, *p* < 0.001) and both of its scales (cognitive-emotional impairment: *r =* 0.24, *p* < 0.001; functional impairment: *r* = 0.15, *p* = 0.006), both types of climate action (individual: *r* = 34, *p* < 0.001; collective: *r* = 0.37, *p* < 0.001), and climate knowledge (*r* = 0.15, *p* = 0.006); it exhibited small but significant positive correlations with depression (*r =* 0.11, *p* = 0.047) and stress (*r =* 0.11, *p* = 0.034). Climate change anxiety was positively associated with both types of climate action, although the correlation was larger for collective action (*r =* 0.46, *p* < 0.001) than individual action (*r* = 0.13, *p* = 0.021).

### 3.3. Path analysis

**Figure 1:**
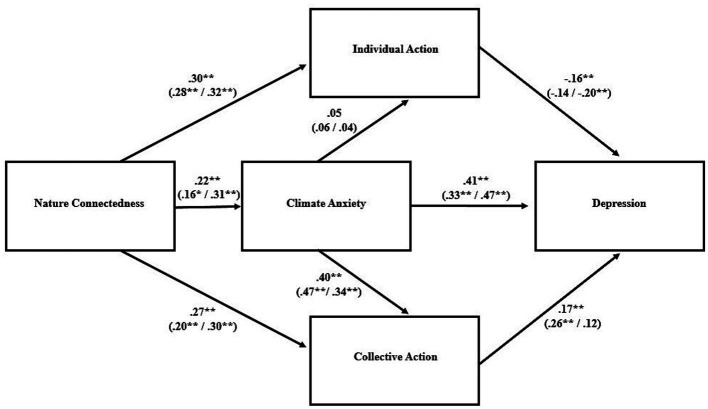
Path model for depression as outcome variable. Standardized parameter estimates are presented for the overall model and, in parentheses, for the low (*left*) and high (*right*) climate knowledge groups. ^**^p < 0.001, ^*^p < 0.05.

**Figure 2:**
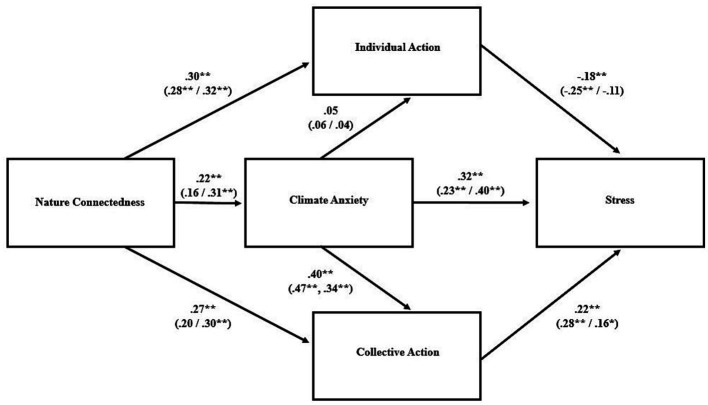
Path model for anxiety as outcome variable. Standardized parameter estimates are presented for the overall model and, in parentheses, for the low (*left*) and high (*right*) climate knowledge groups. ^**^p < 0.001, ^*^p < 0.05.

**Figure 3:**
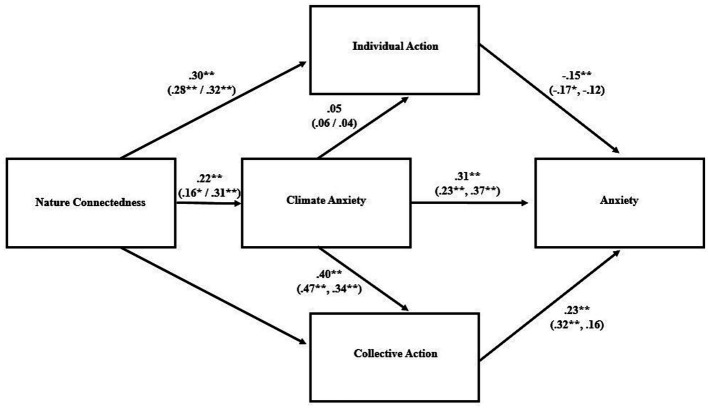
Path model for stress as outcome variable. Standardized parameter estimates are presented for the overall model and, in parentheses, for the low (*left*) and high (*right*) climate knowledge groups. ^**^p < 0.001, ^*^p < 0.05.

Indices related to goodness of fit for the proposed path models are shown in Table 3; the models and their parameter estimates are visualized in Figures 1–3. For the overall sample, the model was an adequate fit for the data for each of the three psychological distress variables. The standardized parameter estimates (Figures 1–3) for specific relationships between variables within the path models aligned closely with the bivariate correlations described above (Table 2).

**Table 3:** Goodness-of-fit indices for path models.

Outcome variable		CFI	TLI	SRMR	RMSEA
Depression	Total	0.99	0.90	0.03	0.07
	High knowledge	0.99	0.94	0.04	0.06
	Low knowledge	0.98	0.86	0.04	0.08
Anxiety	Total	0.98	0.90	0.03	0.07
	High knowledge	0.99	0.92	0.04	0.07
	Low knowledge	0.97	0.79	0.04	0.09
Stress	Total	0.98	0.88	0.03	0.07
	High knowledge	0.99	0.93	0.04	0.06
	Low knowledge	0.96	0.74	0.04	0.10

CFI, Comparative Fit Index; TLI, Tucker Lewis Index; SRMR, Standardized Root Mean Square Residual; RMSEA, Root Mean Square Error of Approximation.

**Figure 4:**
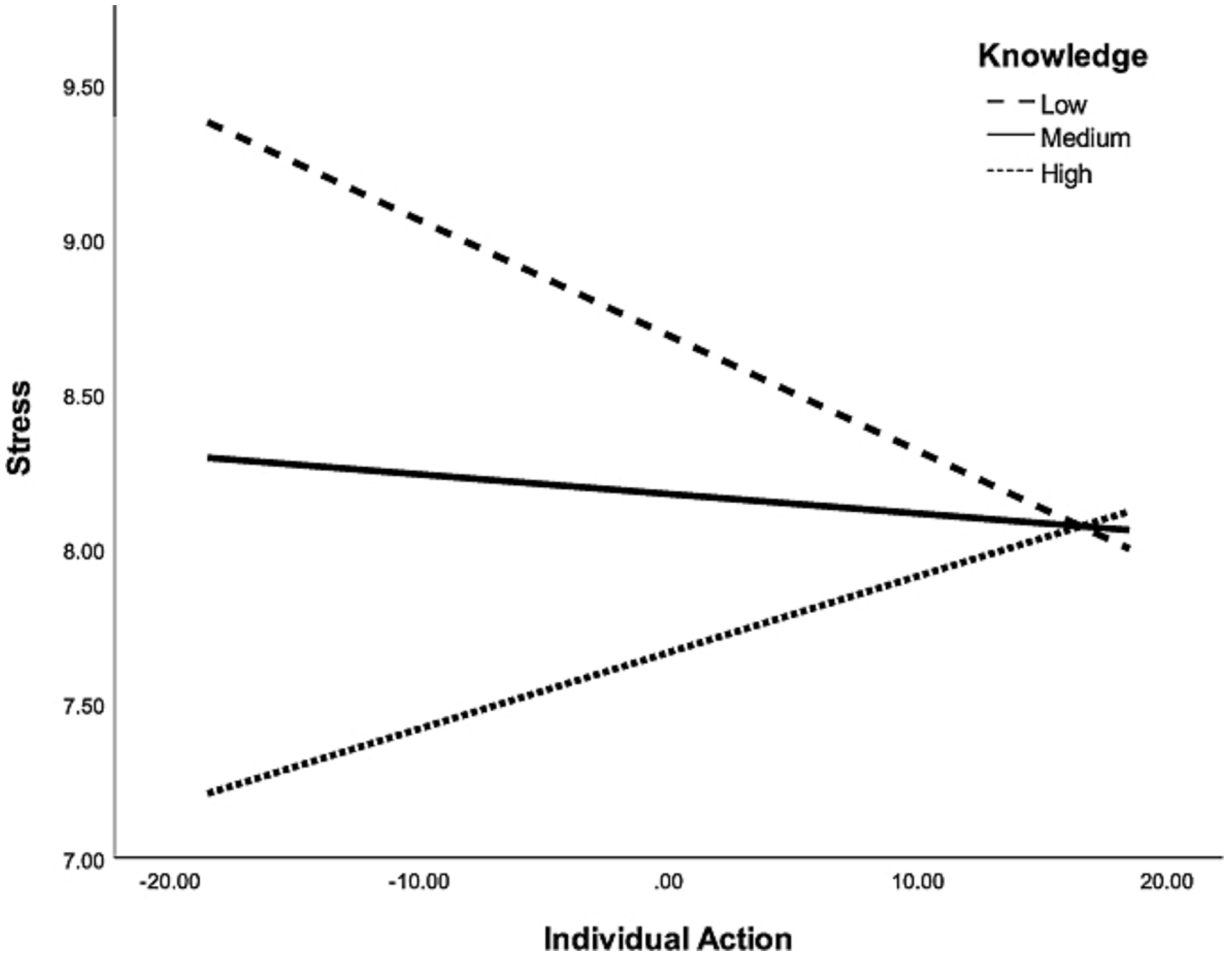
Graphical representation of the significant moderation effect of climate knowledge on the relationship between individual climate action and stress. The lines represent the association at low (−1 SD), medium, and high (+1 SD) levels of climate knowledge.

When the path analyses were conducted again in sub-samples representing high and low climate knowledge, the degree to which the model fit the data varied between groups. In particular, the model was a better fit for the data in the high-knowledge group, as indicated by the indices of fit; in the low-knowledge groups, some of those indicators were outside the range thought to represent an adequate fit (Table 3). Although none of the bivariate correlations between variables were significantly different between the two groups, a few approached significance: the positive association between nature connectedness and climate change anxiety was higher in the high knowledge group (*r* = 0.33) than the low knowledge group (*r* = 0.15; Fisher’s *z* = −1.68, *p* = 0.092), and the positive association between collective action and anxiety was stronger in the low knowledge group (*r* = 0.39) compared to the high knowledge group (*r* = 0.20; Fisher’s *z* = 1.83, *p* = 0.067). Moderation analyses with climate knowledge treated as a continuous variable revealed that knowledge moderated the relationship between individual climate action and stress, such that there was a stronger negative association between them in those with low climate knowledge (Figure 4; Supplementary Table S1). Climate knowledge was not a statistically significant moderator for any of the other bivariate relationships contained within the path model.

The path analyses described above were also conducted using each of the climate anxiety scale’s subscales, cognitive-emotional and functional impairment. For both the overall sample and the sub-samples representing high and low climate knowledge, the goodness of fit indices for the two subscales were very similar to each other and to the results using the full scale (Supplementary Tables S2, S3).

## 4. Discussion

Despite a markedly different climate context in terms of experiencing acute severe climate events, the results were very similar to those found by Curll et al. (2022) in the wake of severe bushfires. As in that case, the proposed path model was an adequate fit for the data, with small, positive associations between nature connectedness and psychological distress that, in our study, were significant in the cases of depression and stress but not anxiety. Similar to Curll et al. (2022), nature connectedness was positively associated with climate change anxiety, which was associated with psychological distress. Nature connectedness was positively associated with both individual and collective climate action. Further mirroring their findings, individual action was negatively associated with psychological distress whereas collective action was positively associated with it.

Thus, overall, the relationships seen here were much like those reported by Curll et al. (2022), despite the geographical difference and the absence of any acute event related to climate change. This supports the generalizability of their findings and highlights the global nature of climate change and its impacts. This is not surprising, given that anxiety and negative emotional responses related to climate change have been reported across a large number of different countries (Hickman et al., 2021; Ogunbode et al., 2022), as has a negative association between such climate-related anxiety and overall mental health (Ogunbode et al., 2022). Likewise, pro-environmental behavior has been positively linked to both climate anxiety (Ogunbode et al., 2022) and nature connectedness (Whitburn et al., 2020) in most countries in which those relationships have been tested. The alignment between the results of the present study and those of Curll et al. (2022) therefore fits with the widespread associations between many of the factors considered here and highlights the widespread impacts of climate change.

The path model that we tested was unique in its inclusion of climate change anxiety, an increasingly well understood construct in terms of its relationship to factors such as pro-environmental behavior (Innocenti et al., 2021) and the impacts of climate change upon mental health (Reyes et al., 2021). Scores on the Climate Change Anxiety Scale (mean for the full scale = 1.94) indicate relatively low levels of climate change anxiety that fall within the range of scores reported elsewhere, which vary widely from 1.25 in a United Kingdom sample (Whitmarsh et al., 2022) to over 2.50 in in an Indian sample (Tam et al., 2023). The scores here were higher than in other North American samples (Clayton and Karazsia, 2020; Schwartz et al., 2022), even immediately following serious heat waves (Bratu et al., 2022). Considering that several studies report that climate change anxiety is higher in young people (Clayton and Karazsia, 2020; Larionow et al., 2022; Whitmarsh et al., 2022), the high scores seen here may be due to our predominantly young sample (81% of participants were under 30 years of age). The restricted range of ages in the present study’s sample also explains the absence of an association between climate change anxiety and age, as documented by the above-mentioned studies.

Reported levels of climate change anxiety in the present study were lower than those of climate worry reported by Curll et al.’s (2022) sample, a difference that aligns with research that directly compares the two (Whitmarsh et al., 2022). A lower prevalence of climate change anxiety compared to mere worry was expected since climate change anxiety involves a more substantial impact on one’s daily functioning, similar to the difference between worrying in general compared to having an anxiety disorder (Clayton and Karazsia, 2020). While the proposed model was an adequate fit to the data overall, there were some noteworthy differences compared to Curll et al.’s (2022) results that are attributable to the above-described difference between climate worry and climate change anxiety. For example, the association of psychological distress with climate change anxiety here was much stronger than its association with climate worry in that study.

Interestingly, the relationship between pro-environmental behavior and climate change anxiety was similar to that seen by Curll et al. (2022) using climate worry, but only in regard to collective action. In the case of individual action, the association with climate change anxiety seen here was much weaker. Positive affect predicts the kinds of behaviors that comprise individual action (Coelho et al., 2017), such that the presence of climate change anxiety, with its substantial negative impact and association with broader psychological distress, is less likely to induce individual action. In contrast, collective climate action has most often been linked to negative emotions (Mallett, 2012; Harth et al., 2013), which aligns with the comparatively strong link with climate change anxiety seen here.

There are many other factors that are likely to influence the relationships examined here; for example, others have demonstrated links between personality traits and engagement in pro-environmental behavior (Brick and Lewis, 2016; Ucar et al., 2023). Here, we assessed whether the observed relationships were influenced by knowledge about climate change. Among those who scored highly (above 60%) on the questionnaire measuring climate knowledge, the proposed path model was an adequate fit for the data. In contrast, the model was a less good fit among those exhibiting low climate knowledge, with indices closer to and in some cases below the values indicating adequate fit. This finding suggests that climate knowledge influences the relationships among the variables included in the model. Given that climate knowledge was not a significant moderator for most of the bivariate relationships within the model, its impact may be dispersed across several relationships contained therein. Future work is needed to fully understand how such relationships differ in relation to climate knowledge.

Nonetheless, there were a few specific relationships within the model for which the difference between high and low climate knowledge approached significance. One was the association between nature connectedness and climate change anxiety, which was stronger among those with high climate knowledge. Moser (2007) argued that some people exhibit apathy or engage in willful repression in order to avoid negative emotions related to climate change. This aligns with the negative association between climate/environment knowledge and climate change anxiety seen both here and in a recent study by Zacher and Rudolph (2023), as well as the association between information seeking and climate change anxiety reported by Whitmarsh et al. (2022). Thus, it may be that, through a lack of such knowledge, some can enjoy their connection to nature in a way in a way that is not spoiled by global issues related to climate and environment.

One of Curll et al.’s (2022) most striking findings was that individual climate action was associated with less psychological distress, whereas collective action was associated with more. This difference, replicated in the present study, corresponds with the findings of a recent meta-analysis that found individual climate actions to be more strongly associated with subjective well-being than collective action (Zawadzki et al., 2020). A recent study by Capstick et al. (2022) found that collective action was associated more strongly with well-being in collectivistic cultures than in individualistic cultures of the type studied here and by Curll et al. (2022). These differences, like those related to climate change anxiety discussed above, may relate in part to the negative emotions (e.g., guilt and anger) associated with engagement in climate action (Van Zomeren et al., 2008; Harth et al., 2013), whereas individual action has been linked to positive emotions such as a greater sense of meaning in life (Jia et al., 2021).

The associations between collective action and psychological distress were stronger among those with low climate knowledge for all three outcome variables, bordering on significance in the case of anxiety. This may reflect differences in the motivations behind individuals’ engagement in collective climate action, a possibility worth addressing in follow-up studies. Considering the potential to target knowledge about climate change as a modifiable factor for boosting pro-environmental behavior, future work should expand upon the present findings to better understand its role.

There were some limitations to the present study. The sample was mostly young people and primarily female, which limits the generalizability of the present findings. The total sample was acquired via two different sample methods (undergraduate student population and general public), creating an age distribution that was mostly young people yet also had featured many people in their 30s and 40s, and beyond; it is possible that this heterogeneity influenced or obscured some relationships. The overall sample size was sufficient for the overall path analysis, it may have restricted our ability to detect more specific differences. This is especially true regarding the role of climate change knowledge, where future work is required to better understand its influence on the link between nature connectedness and climate change anxiety, among other associations. Thus, follow-up work would benefit from acquiring larger sample sizes through singular sampling methods.

While the study was conducted in a geographical location where experiences with acute severe climate events have been very rare, it is nonetheless possible that participants varied in their personal experiences in this regard. Thus, future studies should screen for or measure participants’ individual experiences with climate events. This is especially true in the eastern Canadian region studied here, where some unprecedented weather events occurred in the summer immediately following data collection. Lastly, not measuring climate worry alongside climate change anxiety prevented us from directly comparing how these two related constructs were related to the other factors measured. Future studies exploring such relationships would benefit from including both.

## 5. Conclusion

The present study contributes to our understanding of individuals’ mental health in the context of climate change and their responses to it, exploring the relationships among nature connectedness, climate change knowledge, mental health, and pro-environmental behavior. In replicating the findings of a similar study that was conducted in the wake of severe climate events (Curll et al., 2022), but in a population that has not been impacted in that manner, the present studies suggest that the relationships among these constructs do not depend on context (i.e., the degree to which people have been directly affected by acute climate events) and may be global in nature. This aligns with previous research suggesting global impacts (Hickman et al., 2021) and highlights the need for attention to the mental health effects of climate change everywhere and not just in the wake of acute crises.

The results have some important practical implications in terms of potential engagement of individuals in responding to climate change. The positive association between nature connectedness and both individual and collective climate action raises the possibility that pro-environmental behaviors could be increased through enhancement of nature connectedness. While many studies have shown that brief interventions, often involving some combination of exposure to natural environments and mindfulness trainings, enhance nature connectedness, there is a relative lack of information on how long those increases last and how to induce lasting changes (for review, see Sheffield et al., 2022). At the same time, nature connectedness was associated with climate anxiety and, in turn, higher levels of psychological distress. Thus, any efforts to enhance nature connectedness as a tool for increasing climate action must also take into consideration the potential negative mental health effects. While further work is needed to fully understand the role of climate knowledge within these relationships, some of the present findings (e.g., the stronger link between nature connectedness and climate change anxiety among those with high climate knowledge), further highlight the need to consider possible interventions such as education and enhancement of natural connectedness in a broad scope that considers mental health impacts.

## Data availability statement

The raw data supporting the conclusions of this article will be made available by the authors, without undue reservation.

## Ethics statement

The studies involving humans were approved by Research Ethics Board, University of New Brunswick. The studies were conducted in accordance with the local legislation and institutional requirements. The participants provided their written informed consent to participate in this study.

## Author contributions

ET: Conceptualization, study design, data collection, data analysis, and writing (thesis and manuscript). SR: Conceptualization, study design, data collection, data analysis, writing, and supervision. All authors contributed to the article and approved the submitted version.
