# Supplementary material for: The relationships among nature connectedness, climate anxiety, climate action, climate knowledge, and mental health
Source: Front Psychol. 2023 Nov 15;14:1241400. doi: 10.3389/fpsyg.2023.1241400 (PMC10684686; doi:10.3389/fpsyg.2023.1241400)
Supplement: Supplementary file 1 [file Data_Sheet_1.docx]

Table S1. Results of moderation analysis predicting the outcome variable stress.

| Predictor variable | Coefficient | Standard Error | t | p-value |
| --- | --- | --- | --- | --- |
| Individual action | 0.006 | 0.016 | 0.395 | 0.693 |
| Climate knowledge | 2.956 | 1.737 | 1.701 | 0.090 |
| Action x knowledge | 0.179 | 0.084 | 2.133 | 0.034 |
| Age | 0.011 | 0.035 | 0.316 | 0.752 |
| Gender | 0.306 | 0.346 | 0.883 | 0.378 |

R-squared = 0.029

Table S2. Goodness-of-fit indices for path models using the cognitive-emotional impairment subscale of the Climate Change Anxiety scale.

| Outcome variable | | CFI | TLI | SRMR | RMSEA |
| --- | --- | --- | --- | --- | --- |
| Depression | Total | 0.99 | 0.90 | .03 | .07 |
|  | High knowledge | 0.99 | 0.95 | .04 | .05 |
|  | Low knowledge | 0.97 | 0.82 | .04 | .09 |
| Anxiety | Total | 0.98 | 0.90 | .03 | .07 |
|  | High knowledge | 0.99 | 0.94 | .04 | .06 |
|  | Low knowledge | 0.96 | 0.75 | .04 | .10 |
| Stress | Total | 0.98 | 0.88 | .03 | .07 |
|  | High knowledge | 0.99 | 0.95 | .04 | .05 |
|  | Low knowledge | 0.96 | 0.72 | .05 | .10 |

CFI = Comparative Fit Index; TLI = Tucker Lewis Index; SRMR = Standardized Root Mean Square Residual; RMSEA = Root Mean Square Error of Approximation

Table S3. Goodness-of-fit indices for path models using the functional impairment subscale of the Climate Change Anxiety scale.

| Outcome variable | | CFI | TLI | SRMR | RMSEA |
| --- | --- | --- | --- | --- | --- |
| Depression | Total | 0.98 | 0.88 | .03 | .08 |
|  | High knowledge | 0.99 | 0.94 | .04 | .06 |
|  | Low knowledge | 0.97 | 0.80 | .04 | .09 |
| Anxiety | Total | 0.98 | 0.88 | .03 | .07 |
|  | High knowledge | 0.99 | 0.95 | .04 | .05 |
|  | Low knowledge | 0.96 | 0.70 | .04 | .10 |
| Stress | Total | 0.98 | 0.86 | .03 | .08 |
|  | High knowledge | 0.99 | 0.95 | .04 | .05 |
|  | Low knowledge | 0.95 | 0.67 | .05 | .11 |

CFI = Comparative Fit Index; TLI = Tucker Lewis Index; SRMR = Standardized Root Mean Square Residual; RMSEA = Root Mean Square Error of Approximation
